# Compensatory intestinal immunoglobulin response after vancomycin treatment in humans

**DOI:** 10.1080/19490976.2021.1875109

**Published:** 2021-01-21

**Authors:** Torsten P. M. Scheithauer, Guido J. Bakker, Maaike Winkelmeijer, Mark Davids, Max Nieuwdorp, Daniël H. van Raalte, Hilde Herrema

**Affiliations:** a Department of Experimental Vascular Medicine, Amsterdam UMC, Location AMC at University of Amsterdam, Amsterdam, The Netherlands; b Department of Internal Medicine, Diabetes Center, Amsterdam UMC, Location VUmc at Vrije Universiteit Amsterdam, Amsterdam, The Netherlands

**Keywords:** Immunoglobulin, gut microbiota, LPS, flagellin, vancomycin

## Abstract

Intestinal immunoglobulins (Ig) are abundantly secreted antibodies that bind bacteria and bacterial components in the gut. This binding is considered to accelerate bacterial transit time and prevent the interaction of potentially immunogenic compounds with intestinal immune cells. Ig secretion is regulated by alterations in gut microbiome composition, an event rarely mapped in an intervention setting in humans. Here, we determined the intestinal and systemic Ig response to a major intervention in gut microbiome composition. Healthy humans and humans with metabolic syndrome received oral vancomycin 500 mg four times per day for 7 days. Coinciding with a vancomycin-induced increase in Gram-negative bacteria, fecal levels of the immunogenic bacterial components lipopolysaccharide (LPS) and flagellin drastically increased. Intestinal antibodies (IgA and IgM) significantly increased, whereas peripheral antibodies (IgG, IgA, and IgM) were mostly unaffected by vancomycin treatment. Bacterial cell sorting followed by 16S rRNA sequencing revealed that the majority of Gram-negative bacteria, including opportunistic pathogens, were IgA-coated after the intervention. We suggest that the intestinal Ig response after vancomycin treatment prevents the intrusion of pathogens and bacterial components into systemic sites.

## Introduction

Gut microbial homeostasis is critical to prevent the colonization of potentially disease-causing microbes. The microbiota thereby provides an important line of defense against pathogens. Intestinal homeostasis is in part governed by immunoglobulins (Ig)^1^, which are abundantly secreted from locally residing B cells.^2^ IgA and IgM are mainly present at mucosal sites whereas IgG is the most abundant Ig in the circulation. A substantial fraction of commensals as well as potentially pathogenic bacteria in the intestine is coated with Igs.^2^ This coating accelerates the transit of intestinal bacteria and limits interaction with the intestinal epithelium as well as intestinal immune cells. In addition, Igs can bind and neutralize bacterial components with immunogenic properties such as endotoxin and flagellin. These bacterial components have been suggested to play a causal role^3,4^ in the activation of low-grade inflammation as observed in diseases like type 2 diabetes (T2D).^5^

The Gram-negative bacterial cell wall component lipopolysaccharide (LPS) has been particularly studied in this context as LPS-treatment induces a diabetes-like phenotype in mice.^3^ Although LPS was higher in the circulation of T2D compared to non-diabetic humans,^6^ the contribution of LPS to this disease in humans is controversial.^7^ Flagellin is part of the bacterial locomotor appendage flagellum,^8^ which is critical for colonization of mucosal surfaces and a hallmark of bacterial pathogenicity. Although flagellin has not been as abundantly studied in T2D in humans, it has high immunogenic properties^9^ as shown in gastrointestinal diseases.^10^ Neutralization of these toxins by secreted Igs is of critical importance^11^ but rarely studied in humans.

IgA production by B cells is modified by the gut microbiota^2^ as well as by the above-mentioned bacterial components.^10^ The intestinal Ig response to commensal bacteria has been postulated to be generally polyreactive,^11^ meaning that an antibody is reactive to a variety of structurally related antigens. However, the development of highly affine and specific antibodies is vital to keep pathogens under control.^2^ The importance of strain-level variation in the gut microbiota was recently shown to be critical for the regulation of the mucosal immune response in mice.^12^ In humans, the Ig response, including binding to potentially harmful bacteria and bacterial components, to a significant modification in the gut microbiome, has rarely been mapped.

In this work, we studied the intestinal and systemic Ig response to a major intervention in intestinal homeostasis in healthy humans and in humans with metabolic syndrome. The oral, non-absorbable antibiotic vancomycin was used to increase intestinal LPS and flagellin levels and to shift the gut microbiota toward a disease-associated, predominantly Gram-negative composition. These adverse alterations were compensated for by an increased intestinal, but not systemic, Ig response in both healthy and metabolic syndrome participants.

## Results

Intestinal and systemic Ig response to vancomycin treatment was assessed in 10 healthy lean men and in 10 obese men with metabolic syndrome, all of Caucasian descent. Both groups received a common clinical dose of 500 mg vancomycin, four times per day for 7 days. Of importance, age differed significantly between the groups (**Table S1**). Since age is considered a major confounder in gut microbiota studies^13^ and likely to drive differences between our study groups, we decided to not compare results between lean and obese participants. Instead, we provide data on the overall vancomycin response (groups combined) and on vancomycin response within each group.

### Intestinal Immunoglobulins increase significantly after Vancomycin Treatment

**Figure 1.**
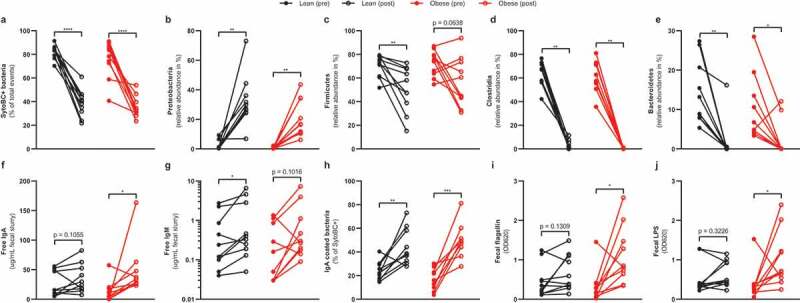
Intestinal changes after vancomycin treatment

Vancomycin treatment strongly decreased bacterial numbers as deduced from reduced nucleic acid staining of fecal bacteria (Figure 1a) and fecal DNA content (Table 1). As anticipated based on vancomycin`s specificity toward Gram-positive bacteria,^14^ the abundance of Gram-negative bacteria such as Proteobacteria (Figure 1b), significantly increased after 7 days of treatment (Table 1).

**Table 1.** Intestinal and serum changes after 1 week of vancomycin treatment

	Overall			Lean			Obese		
	*Pre*	*Post*	*p-value*	*Pre*	*Post*	*p-value*	*Pre*	*Post*	*p-value*
Fecal bacteria (SytoBC+)	**79.3 (12.0)**	**36.9 (10.1)**	**<0.0001**	**82.4 (5.8)**	**38.4 (11.4)**	**<0.0001**	**76.1 (15.7)**	**35.4 (9.0)**	**<0.0001**
Fecal DNA (ug/uL)	**3.38 (1.2)**	**0.66 (0.3)**	**<0.0001**	**3.78 (1.1)**	**0.77 (0.28)**	**<0.0001**	**2.99 (1.2)**	**0.56 (0.36)**	**0.0001**
Fecal IgA (ug/mL)	**20.8 (20.3)**	**42.1 (35.4)**	**0.0053**	**26.7 (21.4)**	**34.9 (24.2)**	**0.1055**	**14.1 (17.4)**	**50.1 (44.7)**	**0.0195**
Fecal IgM (ug/mL)	**0.55 (0.8)**	**1.49 (2.3)**	**0.0054**	**0.70 (1.0)**	**1.58 (2.3)**	**0.0195**	0.40 (0.5)	1.49 (2.4)	0.1055
Fecal flagellin (OD620)	**0.39 (0.4)**	**0.89 (0.6)**	**0.0027**	**0.45 (0.4)**	**0.68 (0.5)**	**0.1309**	**0.33 (0.4)**	**1.11 (0.7)**	**0.0273**
Fecal LPS (OD620)	**0.45 (0.4)**	**0.83 (0.6)**	**0.0153**	**0.52 (0.4)**	**0.64 (0.3)**	**0.3223**	**0.39 (0.4)**	**1.01 (0.7)**	**0.0371**
Fecal IgA anti-flagellin (OD450)	0.59 (0.69)	0.83 (1.11)	0.9843	0.63 (0.74)	0.71 (1.22)	0.4316	0.54 (0.67)	0.95 (1.04)	0.4961
Gram-negative bacteria (%)	**18.5 (11.4)**	**84.1 (13.0)**	**<0.0001**	**20.6 (11.2)**	**84.3 (11.2)**	**<0.0001**	**16.3 (11.7)**	**83.8 (15.3)**	**<0.0001**
IgA+ bacteria (% of SytoBC+)	**20.5 (10.0)**	**46.7 (17.9)**	**<0.0001**	**23.9 (7.8)**	**44.2 (15.1)**	**0.0041**	**16.8 (10.3)**	**49.6 (21.1)**	**0.0005**
CRP (mg/L)	2.22 (3.0)	1.75 (1.9)	0.4619	1.08 (2.1)	0.68 (0.8)	0.9999	3.35 (3.5)	2.82 (2.0)	0.3164
Serum IgG (mg/mL)	**8.55 (3.2)**	**10.32 (4.2)**	**0.0168**	8.31 (2.8)	9.21 (2.9)	0.1934	8.78 (3.7)	11.4 (5.0)	0.1055
Serum IgA (mg/mL)	2.99 (1.8)	3.50 (1.9)	0.1105	2.79 (1.6)	3.09 (1.9)	0.2368	3.21 (2.1)	3.96 (2.0)	0.2480
Serum IgM (mg/mL)	2.12 (2.0)	2.64 (2.3)	0.2268	2.20 (2.4)	2.35 (2.3)	0.5566	2.03 (1.6)	2.93 (2.4)	0.3223
Serum IgG anti-flagellin (OD450)	1.80 (0.8)	1.82 (0.8)	0.9215	1.45 (0.5)	1.40 (0.5)	0.8290	2.14 (0.87)	2.24 (0.9)	0.8013
Serum IgA anti-flagellin (OD450)	1.39 (0.7)	1.33 (0.77)	0.7812	1.16 (0.7)	1.21 (0.8)	0.8842	1.65 (0.7)	1.46 (0.8)	0.5361
Serum IgM anti-flagellin (OD450)	1.00 (0.6)	1.07 (0.6)	0.6980	1.12 (0.6)	1.10 (0.6)	0.9118	0.88 (0.6)	1.03 (0.6)	0.5827
Serum flagellin (OD620)	0.16 (0.05)	0.18 (0.07)	0.4831	0.16 (0.04)	0.17 (0.06)	0.8148	0.17 (0.05)	0.19 (0.07)	0.5040
Serum LPS (EU/mL)	**1.04 (0.96)**	**2.73 (2.22)**	**0.0002**	**0.99 (0.58)**	**2.91 (2.31)**	**0.0137**	**1.09 (1.26)**	**2.55 (2.23)**	**0.0117**

Lean healthy and obese people with metabolic syndrome were treated with vancomycin for seven days. The intestinal and serum antibody response as well as the microbial activity was measured before and after treatment. The mean (standard deviation) results are shown. Statistical analysis was performed using paired t-test or Wilcoxon signed-rank test.

Healthy lean (n = 10) and obese people with metabolic syndrome (n = 10) were given vancomycin for 7 days. The total amount of intestinal bacteria decreased after treatment (a). Vancomycin treatment increased Proteobacteria (b) but decreased Firmicutes (c), Clostridia (d), and Bacteroidetes (e). Lastly, it increased intestinal free IgA (f), IgM (g), IgA-coated bacteria (h), bacterial flagellin (i), lipopolysaccharide (j). Statistical analysis was performed using paired t-test (a, c, h) or Wilcoxon signed-rank test (b, d – g, j, i): *p < .05, **p < .01, ***p < .001, ****p < .0001.

Despite predominant activity against Gram-positive bacteria, vancomycin affects Gram-negative bacteria as well.^15^ Indeed, in addition to a depletion of Gram-positive bacteria belonging to Firmicutes (mainly Clostridia) (Figure 1c,d, respectively), a substantial part of Gram-negative Bacteroidetes (Figure 1e) was depleted as well.^14^ Thereby, vancomycin significantly alters the prevalence of members of the main phyla of the human gut microbiota.^16^

Fecal IgA and IgM (Table 1, Figure 1f,g) as well as the number of IgA-coated bacteria increased after vancomycin treatment (Table 1, Figure 1h). Of note, participant #12 appeared IgA deficient. It is known that IgA deficiency can be compensated for by IgM.^17^ Indeed, participant #12 had one of the highest IgM levels before (1.13 ug/mL) and the highest level after vancomycin (7.32 ug/mL). We decided to keep this participant in the cohort since he was asymptomatic and we included the overall intestinal antibody response, not only IgA, during antibiotic treatment.

In line with a predominantly Gram-negative bacterial composition upon treatment, fecal levels of bacterial toxins flagellin (Table 1, Figure 1i) and LPS (Table 1, Figure 1j) increased. Although plasma levels of IgA and IgM were unaffected by vancomycin treatment, plasma levels of IgG moderately increased (Table 1).

### Serum bacterial Toxins positively correlate with intestinal bacterial toxins and increase during high-fat meal test

**Figure 2.**
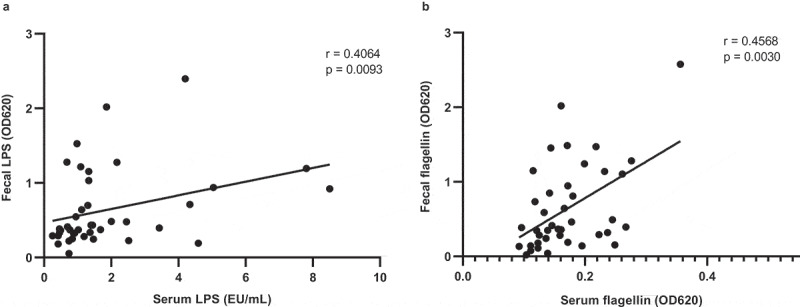
Association between fecal and serum LPS as well as flagellin

We previously reported that vancomycin increases serum LPS concentrations (Table 1).^15^ We here show that serum LPS concentrations positively correlate with fecal LPS concentrations (Figure 2a). In addition, serum and fecal flagellin concentrations strongly correlated (Figure 2b). Despite a massive increase in fecal flagellin, serum flagellins did not increase (Table 1). Further, Igs against this bacterial component were unaffected in both serum and feces (Table 1).

Healthy lean (n = 10) and obese people with metabolic syndrome (n = 10) were given vancomycin for 7 days. Fecal and serum LPS (a) as well as flagellin (b) positively correlate. Statistical analysis was performed using the non-parametric Spearman correlation (a, b).

**Figure 3.**
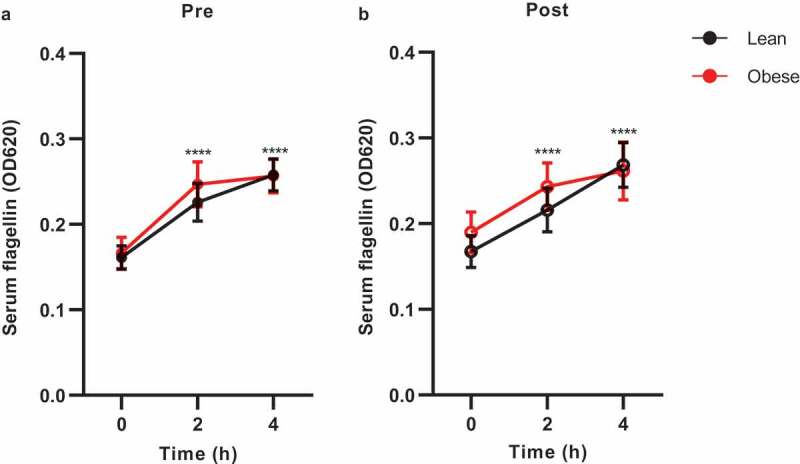
Serum flagellin concentrations during the high-fat meal

Several routes of bacterial translocation have previously been postulated.^18^ Translocation has been proposed to occur particularly after ingestion of a high-fat meal. Indeed, this so-called postprandial inflammation has been associated with increased serum LPS levels after a high-fat meal.^19^ Although we were unable to reproduce these findings for LPS,^15^ serum flagellin levels were significantly increased 2 and 4 hours after ingestion of a high-fat meal (Figure 3a,b). Of interest, this effect was not altered by vancomycin treatment (Table 1 and **Table S2**), despite increased fecal flagellin levels.

Healthy lean (n = 10) and obese people with metabolic syndrome (n = 10) were given vancomycin for 7 days. Serum flagellin increased during the high-fat meal before (a) and after (b) vancomycin treatment. Mean with SEM is shown. Statistical analysis was performed using two-way repeated measures ANOVA with Bonferroni’s multiple comparison (a, b). Statistical significance indicates a difference to baseline (N = 20). ****p < .0001.

We previously reported that a high-fat meal increases circulating lymphocytes (including antibody-producing B cells) in the obese group.^15^ We, therefore, measured Ig concentrations during the fat meal test. The high-fat diet did not alter the total serum antibody concentrations (**Table S2, Figure S1**).

High-fat meal-induced alterations in systemic Igs against flagellin were inconsistent and highly individual. The overall trend showed reduced IgA and IgG against flagellin, whereas IgM against flagellin increased (**Table S2**) but are inconclusive in this cohort. Vancomycin did not alter those findings. Similarly, antibodies against the flagellin-bearing, Gram-negative bacteria *Escherichia coli*, which was abundantly present after vancomycin treatment, were not significantly affected (**Table S2**). These findings suggest a non-compromised gut barrier function in both study groups.

### The vast majority of bacteria is coated by immunoglobulins after vancomycin treatment

IgA production is modified by the gut microbiota.^2^ To address vancomycin-induced changes in microbial composition and intestinal Ig response in more detail, we sorted IgA-coated and uncoated bacteria followed by 16S rRNA-based characterization of these populations before and after vancomycin treatment.

The aforementioned participant #12 was excluded from hereon as he had undetectable IgA levels and IgA-based sorting could not be performed. Multilevel principal component analysis (PCA) showed a clear separation between positive, negative, and unsorted samples without clustering of groups (**Figure S2**). To test the effect of host phenotype (lean or obese) and vancomycin treatment on IgA coating of specific taxa, we applied linear mixed-effects models (LME) on the top 500 most prevalent and abundant microbes. This analysis revealed that only one amplicon sequence variant (ASV) was differently coated due to host phenotype (*p* = .027, sorting mode x group, **Table S3**). ASV_0915 (*Clostridiales* bacterium DTU089) was significantly more coated with IgA (*p* < .0001) and more abundant in the IgA fraction of obese compared to lean subjects (*p* = .0043).

Since vancomycin had extreme effects on microbiome composition whereas host phenotype was barely associated with IgA coating, we performed separate analysis on both time points and combined groups in the following analyses.

**Figure 4.**
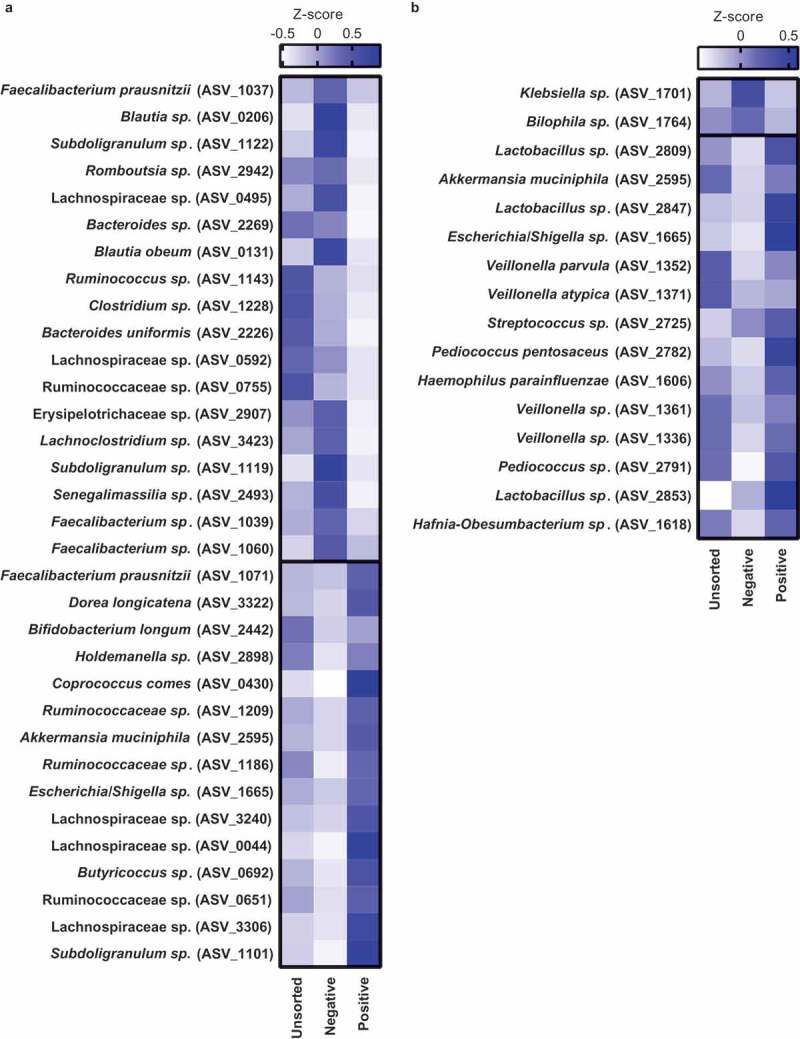
IgA-coated bacteria before and after vancomycin treatment

LME analysis revealed that 260 out of 500 tested ASVs were significantly (*p* < .05) enriched in either IgA positive or negative fraction before vancomycin treatment (**Table S4**) and 148 ASVs after treatment (**Table S5**). Some of these significant ASVs, however, were either present in only a few participants or had a very low abundance. In order to simplify the vast number of significant hits and to highlight biologically relevant bacteria, we next focused on bacteria that were present in at least 60% of the participants and that had an abundance of at least 0.1% (Figure 4).

Healthy lean (n = 10) and obese people with metabolic syndrome (n = 9) were given vancomycin for 7 days. Intestinal bacteria were sorted and sequenced before (A) and after treatment (B). The heatmaps show the most abundant bacteria (>0.1%, present in >60% of the participants) that are significantly (*p* < .05) enriched either in the IgA negative (top) or positive (bottom) fraction. The heatmaps are sorted according to their relative abundance (bottom-to-top per fraction). Z-scores of their abundance are shown. Statistical analysis was performed using the Wilcoxon matched-pairs signed-rank test.

**Figure 5.**
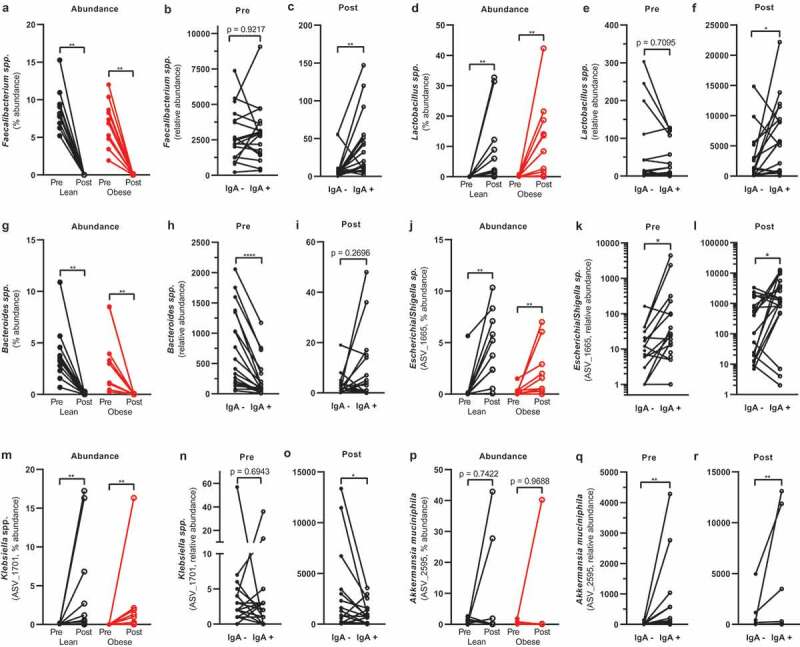
Selection of IgA-coated bacteria. Healthy lean (n = 10) and obese people with metabolic syndrome (n = 10) were given vancomycin for seven days. Vancomycin changed the relative abundance of several intestinal bacteria (a, d, g, j, m, p). Bacteria were differentially coated with IgA before (b, e, h, k, n, q) and after treatment (c, f, i, l, o, r). Statistical analysis was performed using Wilcoxon matched-pairs signed-rank test (a): *p < .05, **p < .01, ****p < .0001

ASVs belonging to the phyla Firmicutes were differentially coated with IgA, suggesting that IgA coating is specific to species or even strains. For example, the butyrate producer *Faecalibacterium prausnitzii* is highly abundant in the human intestine with ratios ranging up to 15% of total bacteria.^20^ In line, the abundance of *Faecalibacterium spp*. ranged from 1.92% to 15.27% (Figure 5a) in our study. The genus *Faecalibacterium* was not enriched in either of the IgA fractions before treatment (Figure 5b). After treatment, it was enriched in the IgA positive fraction (Figure 5c), despite the low abundance at that time point (0.01%). ASVs belonging to the genus *Faecalibacterium* had differential IgA coating profiles (Figure 4a, **Figure S4**), suggesting that IgA coating is species- and strain-specific. Interestingly, two highly abundant ASVs representing *Faecalibacterium prausnitzii* (ASV_1071 and ASV_1037) were differently coated. Before treatment, ASV_1071 was IgA-coated, whereas ASV_1037 was IgA-uncoated. The effect size, which is a reflection of the difference in bacterial abundance in the IgA-coated fraction, was low (**Table S4** and **Table S5**) suggesting weak IgA coating.

Although the Gram-positive genus *Lactobacillus*, belonging to the Firmicutes, has been reported to be IgA-coated,^2^ *Lactobacillus* was not significantly enriched in the IgA positive fraction in our cohort (Figure 5d–e). We speculate this to be due to the low abundance before treatment. After treatment, *Lactobacillus* abundance increased from 0.09% to 11.01% (Figure 5d) and was significantly enriched in the IgA fraction (figure 5f) with several abundant ASVs enriched in the IgA positive fraction (Figure 4b, **Figure S4**). We found a similar enrichment in the IgA positive fraction with *Veillonella* spp. (Figure 4b).

*Bacteroides* are commensal bacteria that perform several beneficial functions for the host. However, when crossing the intestinal wall, they are a known threat to host health.^21^ We, therefore, addressed the IgA coating of *Bacteroides* spp. before and after treatment. *Bacteroides* spp. decreased in both groups after vancomycin treatment (Figure 5g). Although *Bacteroides* spp. was enriched in the negative fraction before treatment (Figure 5h), there was no difference in enrichment after treatment (Figure 5i).

The Gram-negative Proteobacteria increased after vancomycin treatment. The most abundant ASV belonging to Proteobacteria, namely *Escherichia/Shigella sp*. (ASV_1665), increased after treatment (Figure 5j). This ASV was enriched in the IgA positive fraction before (Figure 5k) and after treatment (Figure 5l), which is generally supported by other studies.^2^ However, not all Proteobacteria are IgA-coated. *Klebsiella* spp. (ASV_1701, Figure 5m–o), for example, was enriched in the negative fraction after treatment (Figure 5o) despite a striking increase in abundance after treatment (0.03% to 3.43%).

The Gram-negative *Akkermansia muciniphila* is generally considered to have beneficial effects on host metabolism. *A. muciniphila* (ASV_2595) abundance was not affected by vancomycin treatment (Figure 5p). In line with previous reports,^22,23^ *A. muciniphila* was IgA-coated before (Figures 4a, Figures 5q) and after vancomycin treatment (Figures 4b, Figures 5r) potentially due to its pro-inflammatory potential^24^ and close proximity to the intestinal epithelium.^25^

## Discussion

We here describe in a cohort of healthy and metabolic syndrome participants that short-term vancomycin treatment increases the abundance of Gram-negative opportunistic pathogens and immunogenic bacterial components. This significant adverse alteration in gut microbiota composition was compensated for by a protective intestinal, but not serum, Ig response. Although IgA coating of intestinal bacteria has been previously described,^2,26,27^ we are the first to characterize IgA-coated bacteria before and after antibiotic treatment. We reproduced findings of IgA-targeted bacterial taxa before treatment^22,26,27^ and revealed a novel coating pattern of several opportunistic pathogens. Of interest, some taxa seem to evade the immune response.

In line with previous work, *E. coli^26^* and *A. muciniphila*^23^ were particularly coated with IgA before treatment. Although the IgA coating of these species was well conserved after vancomycin treatment, we observed that the vast majority of bacteria was coated with IgA. We speculate that the significant reduction in bacterial abundance facilitates aspecific IgA binding. Interestingly, IgA-degrading bacteria^28^ such as the Gram-negative *Sutterella wadsworthensis* increased after treatment (ASV_1537, 0.03% to 2.25%). Although increased IgA levels might give these bacteria a niche to bloom, we did not find a significant correlation between this ASV and fecal IgA (Spearman, *p* = .1809, r = 0.2217).

In our cohort, several opportunistic bacteria such as the Proteobacteria *E. coli, Haemophilus parainfluenzae*, and *Pseudomonas aeruginosa* were coated with IgA. Proteobacteria are considered to be IgA-coated because of their inflammatory potential. In contrast, several other opportunistic pathogens in our cohort were not coated with IgA. We can only speculate that proximity to the intestinal wall or specific antigen display on the bacterial cell wall might play a role in this observation. Further, we cannot exclude that low bacterial abundance alters IgA coating. For example, a very high abundance might lead to unspecific IgA coating in the intestine, whereas a low abundance might lead to technical problems in either of the fraction.

IgA coating of several *Lactobacillus* species has been reported in our and several other studies.^22,23^ Lactobacillus was specifically enriched in the IgA-coated fraction after vancomycin treatment. This group of bacteria is intrinsically resistant to vancomycin,^29^ explaining why they survive this harsh intervention. Despite their known beneficial effects on the host,^30^ it is not known why these bacteria are coated with IgA. We propose that this is a relic from birth. Vaginal delivery exposes the infant mainly to Lactobacillus species, which rapidly colonizes the gut and is further supported by milk consumption.^30^ At that stage, the infant’s immune system might develop an immune response against those bacteria to prevent infections, which is maintained for life. Similarly, *Veillonella spp*. was IgA-coated, which is one of the first bacterial groups that colonize the infant’s gut.^13^

Interestingly, antibodies with cross-reactive activity against several IgA-targeted taxa do not bind most Bacteroides *in vivo*.^11^ In fact, bacteria belonging to the Gram-negative Bacteroides have been reported to be collectively uncoated potentially by expressing cell surface molecules that prevent antibody binding.^2^ Indeed, *Bacteroides spp*. was mainly uncoated in our cohort. Nevertheless, *Bacteroides* is of importance for the regulation of Ig production. For example, *Bacteroides ovatus* is able to promote intestinal IgA production.^12^

The significant vancomycin-mediated shift in microbiota composition coincided with an increase in fecal LPS and flagellin levels. In our short-term setting, peripheral LPS increased whereas flagellin levels were not significantly affected after vancomycin treatment. These two different findings might be explained either technically (TLR reporter cell line vs. LAL assay) or by a different structure of these bacterial molecules (protein vs. lipid).

Flagellin facilitates bacterial motility, adhesion to the epithelium, and biofilm formation.^31,32^ The latter is an important bacterial defense system.^33^ Interestingly, *K. pneumoniae*, a common biofilm-forming bacterium that expresses flagella^34^ but circumvents IgA coating in our dataset. In contrast, *E. coli*, also flagellum-bearing, is heavily coated with IgA.^35^ Therefore, the potential to express virulence factors such as flagellin does not necessarily implicate IgA coating. Expression of flagella and surface antigens recognized by IgA depends on environmental factors. In *in vitro* settings, most bacteria display vastly different patterns of surface antigens.^11^ Although our data set did not allow testing of this difference, antibiotic treatment might increase bacterial interaction with intestinal cells and increase the odds for bacterial infections.

Although intestinal antibody production against flagellin was not significantly increased, we speculate that the local antibody response was sufficient to prevent leakage of bacterial toxins into the circulation. LPS^36^ and flagellin^37^ have been shown to induce the production of antimicrobial proteins in the intestine via the expression of Regenerating islet-derived protein 3 (RegIII). Although our experimental setting did not allow us to address this, it is plausible that the increase in intestinal LPS and flagellin after short-term vancomycin treatment induces a similar response in intestinal B cells in humans, thereby, promoting specific antibodies. Interestingly, in a dynamic setting (*i.e*., during a high-fat meal test), serum flagellin levels were increased. This is in support of the concept of postprandial inflammation where bacterial toxins are proposed to enter the circulation together with chylomicrons.^38^

Serum antibodies did not change significantly during the fat meal test, despite the gradual increase in serum flagellin and leukocytes.^15^ We noticed a non-significant drop in total antibodies after treatment in both groups. We can only speculate that these antibodies might bind translocating bacterial components to buffer a pro-inflammatory reaction. However, the power of this study is too small to draw conclusions and a follow-up study with greater power and age-matched groups is warranted. Further, it would be of interest to follow up on these patients to explore how the gut microbiota and the antibody response recover after cessation of vancomycin treatment. Lastly, it is of importance to explore how infections (indications for antibiotic treatment) are affecting the response to antibiotics and how that further changes the Ig response. In the present study, none of the participants showed any signs of infections (CRP levels in the normal range). It is plausible that preexisting infections further increase the intestinal Ig concentrations after antibiotic treatment.

In this study, we report several changes in the gut microbiota and the human Ig response after vancomycin treatment. We recognize that based on the data derived from this human trial, we can only speculate about the mechanisms involved and that dedicated studies are required to unravel the alterations observed. In line, there are many outstanding questions on Ig specificity and regulation.^39^ Although our work provides insight only at the level of binding of Igs to bacteria and bacterial components, we feel our data contribute to much-required studies to reveal functional mechanisms in humans.

## Methods

### Study design

The study was performed as previously described.^15^ Lean (body mass index [BMI] between 18.5 and 25 kg/m^2^) and obese (BMI ≥ 30 kg/m^2^), metabolic syndrome (≥3/6 criteria: waist circumference >102 cm; blood pressure ≥130 mmHg systolic or ≥85 mmHg diastolic; fasting plasma glucose ≥5.6 mmol/L; high-density lipoprotein cholesterol [HDL-C] <1.03 mmol/L; fasting triglycerides ≥1.7 mmol/L; homeostatic model assessment-insulin resistance [HOMA-IR] >2.2) male subjects aged 18–75 years were recruited via local advertisements. Subjects were excluded if they met one of the following criteria: use of any medication; use of antibiotics or proton pump inhibitors in the past 3 months; a medical history of type 1 or 2 diabetes, stroke, myocardial infarction, pacemaker, or cholecystectomy; smoking; use of >5 units of alcohol daily; recreational drug use; use of pre-, pro- or synbiotics. All participants received oral vancomycin 500 mg four times a day for 7 days. High-fat meal tests were scheduled before and after vancomycin intervention. To ensure complete wash-out of the study medication, post-intervention visits were scheduled 2 days after cessation of vancomycin. All participants gave written informed consent and the study was approved by the Institutional Review Board of the Amsterdam UMC, location AMC, the Netherlands (conducted in accordance with the Declaration of Helsinki, version 2013).

### High-fat meal tests

High-fat meal tests were performed as previously described.^15^ In brief, subjects visited the clinical trial unit after an overnight fast. Blood samples were collected before (t = 0 h) and after (t = 2 h and t = 4 h) ingestion of a liquid high-fat meal (fresh cream, 35% fat, Albert Heijn, Zaandam, the Netherlands), containing 335 kcal, 35 g fat, 3 g carbohydrates, and 2.5 g protein per 100 mL. Each participant received 50 g of fat per square meter body surface area.^40^

### Biochemical analysis

Assays were performed as described before.^15^ In brief, blood was collected in Vacutainer® tubes containing a polymer gel for serum separation (Beckton Dickinson, Franklin Lakes, NJ), centrifuged at 1550 x g (15 min, 4°C), and stored at −80°C until further analysis.

### Fecal sample collection analysis

Gut microbial analysis was performed as previously described.^15^ In brief, fecal samples were collected by participants at home, stored at 4°C, transported to the hospital within 24 h, and stored at −80°C until further use.

### Fecal IgA flow cytometry and sorting of IgA+ bacteria

To determine the abundance of intact bacteria before and after vancomycin, fecal bacteria were stained with the nucleic acid stain SytoBC (ThermoFisher Scientific, Invitrogen, Carlsbad, CA) according to Kau et al.^26^ with some modifications. Frozen fecal samples (100 mg) were diluted 1:10 in phosphate-buffered saline (PBS) with cOmpleteTM Protease Inhibitory Cocktail (Roche, Basel, Switzerland). All steps were performed on ice. Samples were homogenized with the aid of a 5 mm stainless steel bead (Qiagen, US). Samples were centrifuged at 400 xg for 5 min to separate the bacteria from large debris. Next, 100 μL of the supernatant was taken and centrifuged for 5 min at 8000 x g to pellet the bacteria. The supernatant was saved for total antibody concentrations (fecal water). The pellet was washed with 1 mL of PBS and stained with 100 uL of 50x diluted goat anti-human IgA conjugated with Dylight650 (polyclonal, Abcam, UK) for 30 min. After a last wash, the bacteria were stained with 4000x diluted SytoBC stain in a 0.9% NaCl solution with 0.1 mol/L HEPES (Gibco, US). Stained bacteria were measured on a BD FACS Canto II (Becton, Dickinson, Franklin Lakes, NJ) and analyzed using FlowJo software (version 10.0 FlowJo, LLC, Ashland, OR). Threshold settings were set to the minimal allowable voltage for side scatter to be able to measure small particles. At least 50.000 events were counted. Gating strategies are found in supplementary **Figure S5**.

Bacterial sorting was performed according to Palm et al.^27^ with some modifications. Fecal bacteria were prepared as described above. Bacteria were washed with 1 mL PBS containing 1% Bovine Serum Albumin (BSA, Sigma-Aldrich, US, staining buffer). Pellets were blocked in 100 uL blocking buffer (20% normal mouse serum in staining buffer, Jackson Immuno Research, UK) for 20 min on ice and then stained with 100 uL PE-conjugated anti-human IgA in staining buffer (1:50; Miltenyi Biotec, clone IS11-8E10, Germany) for 30 min on ice. Samples were washed 3 times with 1 mL staining buffer before cell separation. Anti-IgA stained fecal bacteria were incubated in 1 ml staining buffer containing 50 uL anti-PE magnetic-activated cell sorting (MACS) beads (Miltenyi Biotec, Germany) for 15 min at 4°C. Next, bacteria were washed twice with 1 mL staining buffer (10.000 xg, 5 min, 4°C) and separated with an LS column on a manual separator (Miltenyi Biotec, Germany). Both positive and negative fractions were further purified with SH800 cell sorter (Sony, Japan). For each sample, 2 million bacteria were collected, pelleted (10,000 xg, 5 min, 4°C), and frozen for DNA isolation. Gating strategies are found in supplementary **Figure S7**.

### DNA extraction and sequencing analysis of IgA-coated bacteria

DNA was extracted from 250 mg fecal material and the sorted fractions using a repeated bead-beating protocol^41^ (method 5). DNA was purified using the Maxwell RSC Whole Blood DNA Kit. 16S rRNA gene amplicons were generated using a single-step PCR protocol targeting the V3-V4 region.^42^ PCR products were purified using Ampure XP beads and purified products were equimolar pooled. The libraries were sequenced using a MiSeq platform using V3 chemistry with 2 × 251 cycles.

Forward and reverse reads were truncated to 240 and 210 bases, respectively, and merged using USEARCH.^43^ Merged reads that did not pass the Illumina chastity filter, had an expected error rate higher than 2 or were shorter than 380 bases were filtered. Amplified Sequence Variants (ASVs) were inferred for each sample individually with a minimum abundance of four reads.^44^ Unfiltered reads were then mapped against the collective ASV set to determine the abundances. Taxonomy was assigned using the RDP classifier^45^ and SILVA^46^ 16S ribosomal database V132. Contaminants were identified using decontam software and subsequently, together with lab-specific known contaminants, removed from the data. Raw sequence reads were submitted to the ENA repository under study PRJEB27010.

### Antibody ELISAs

Total antibodies were measured in serum or fecal water (see preparation above) according to the manufacturer’s instructions (Total IgG, IgA, and IgM Human Uncoated ELISA kit with Plates, ThermoFisher Scientific, US).

### Flagellin and LPS detection

Flagellin and LPS were detected in serum and fecal water (see above) with the aid of HEK-blue^TM^ TLR5 or HEK-blue^TM^ TLR4 reporter cell line (InvivoGen, US) according to the manufactures instructions. For serum flagellin detection, 20 uL serum was mixed with 180 uL cell suspension. Serum LPS was measured as described before.^15^

### Bacteria and flagellin specific antibody ELISA

*Escherichia coli* (DSM #5911, DSMZ, Germany) was cultured overnight in LB broth (Miller, Sigma-Aldrich, US). The optical density was measured at 600 nm and bacteria were diluted to 10^9^ cells/mL. Bacteria were pelleted at 8000 x g, 5 min, 4°C, and washed with 1 mL sterile PBS (Fresenius Kabi, The Netherlands). Bacteria in PBS were sonicated for 10 seconds at 30% amplitude and 30 seconds intervals between cycles (20x in total, stored on ice water). Nunc MaxiSorp^TM^ flat-bottom ELISA plates (ThermoFisher Scientific, US) were coated with sonicated bacteria (500x diluted in sterile PBS, 100 uL per well) overnight at 4°C. Plates were washed 3x with 300 uL PBS with 0.05% Tween20 and blocked with 150 uL PBS with 1% BSA for 2 hours at room temperature. Next, plates were washed and loaded with 100 uL of diluted serum samples (100x diluted in PBS with 1% BSA) for 4 hours at room temperature. Plates were washed again and loaded with secondary antibodies for 2 h at room temperature (HRP-conjugated anti-human IgG, BD, clone G18-145, 2500x diluted in PBS with 0.05% Tween20; goat HRP-conjugated anti-human IgA, preadsorbed, Abcam, polyclonal, 10.000x diluted; goat HRP-conjugated anti-human IgM, preadsorbed, polyclonal, Abcam, 50.000x diluted). Plates were washed again and incubated with 100 uL TMB for 15 minutes and stopped with 100 uL 2 M H2SO4. Antibody activity was measured by reading OD450.

Antibodies were measured according to Tran, Ley, Gewirtz and Chassaing.^10^ Ninety-six well half area ELISA microplates (ThermoFisher Scientific, US) were coated with 50 uL flagellin (100 ng per well in 9.6 pH bicarbonate buffer; Invivogen, US) overnight at 4°C. Plates were washed 3x with 150 uL PBS with 0.05% Tween20 and were loaded with 50 uL of diluted serum samples (100x diluted in PBS) for 1 hour at 37°C. Plates were washed again and loaded with secondary antibodies for 1 h at room temperature (same dilution as above). Plates were washed again and incubated with 50 uL TMB for 15 minutes and stopped with 50 uL 2 M H2SO4. Antibody activity was measured by reading OD450.

### Statistical analysis

Data were checked for normality with the Shapiro–Wilk test. Effects of vancomycin on fasting parameters were assessed using the paired t-test for normal continuous variables and the Wilcoxon signed-rank test for other variables. One-way ANOVA for repeated measures for normal continuous variables and the Friedman test for other variables, with Bonferroni post hoc testing, was used to assess the effects of the high-fat meal on postprandial parameters. Two-way repeated measures ANOVA with Bonferroni post hoc testing with time after the meal (time) and treatment (pre- vs post-intervention) as factors was used to determine the effects of treatment (time x treatment interaction) on postprandial parameters. Statistical analyses were performed using GraphPad Prism version 8.0.2. Data are provided as mean with standard error of the mean (SEM). Microbiome data were analyzed and visualized in R^47^ (V3.6.3). Permutation ANOVA from Vegan^48^ was used to test differences in composition. Multilevel PCA was performed, on clr transformed data, using the mixOmics^49^ package, statistical significance was tested using a permutation manova on the first 10 components. Effects of sorting on specific taxa were tested with linear mixed effect models using lme4.^50^ *P*-values were corrected for multiple testing (FDR) were applicable, and *P*-values <0.05 were considered statistically significant. All authors had access to the study data and reviewed and approved the final manuscript.

## Supplementary Material

Supplemental Material

Supplemental Material
