# Supplementary material for: Compensatory intestinal immunoglobulin response after vancomycin treatment in humans
Source: Gut Microbes. 2021 Jan 21;13(1):1875109. doi: 10.1080/19490976.2021.1875109 (PMC7833805; doi:10.1080/19490976.2021.1875109)
Supplement: Supplemental Material [file KGMI_A_1875109_SM6893.docx]

**Supplementary Figures and Tables**


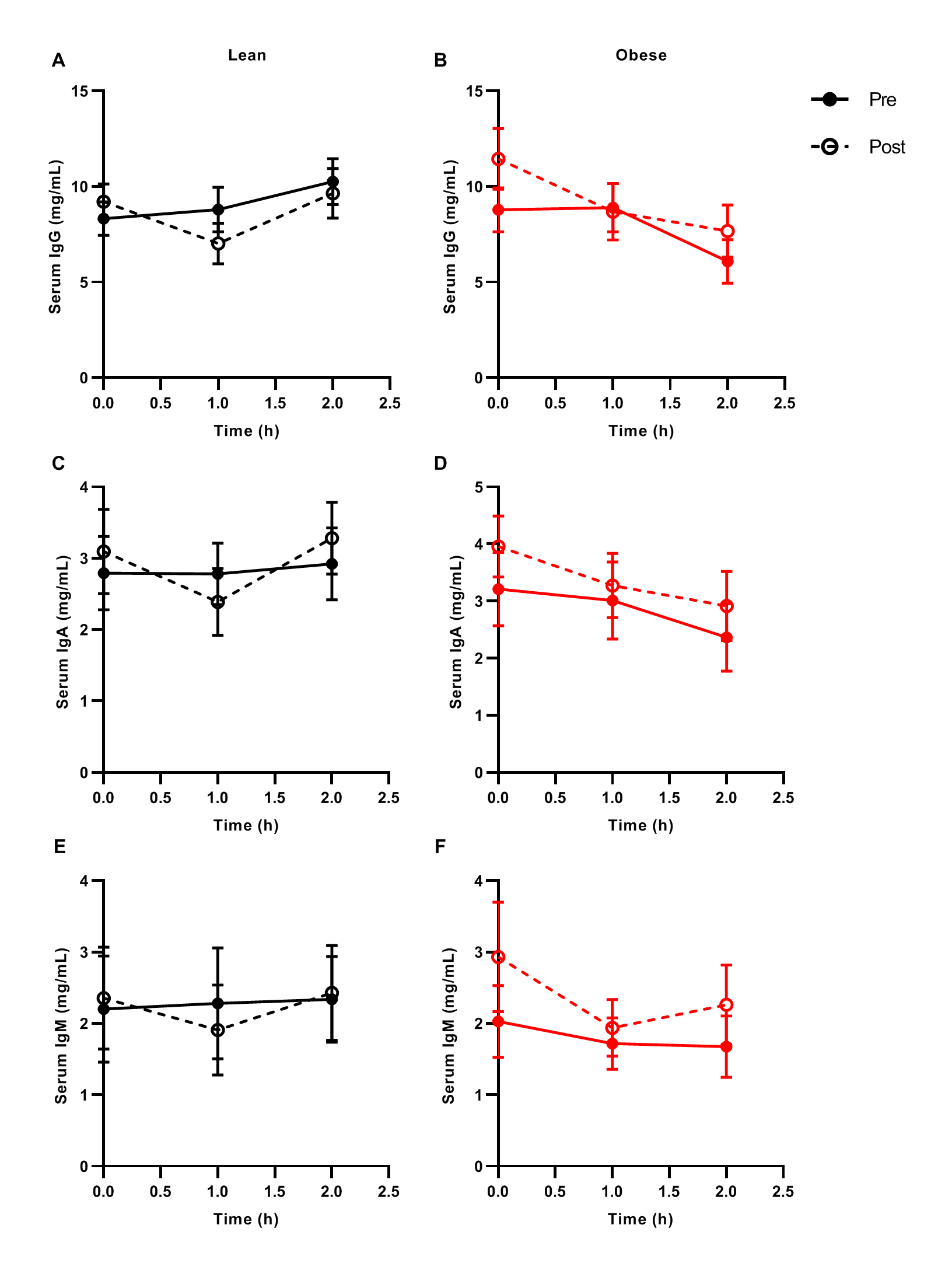


**Figure S1.** Serum antibodies do not change during high fat meal test.

Healthy lean (n = 10) and obese people with metabolic syndrome (n = 10) were given vancomycin for seven days. Serum antibodies did not change during high fat meal before and after vancomycin treatment. Mean with SEM is shown for every antibody type. Lean individuals are on the left (black) and obese individuals on the right (red).


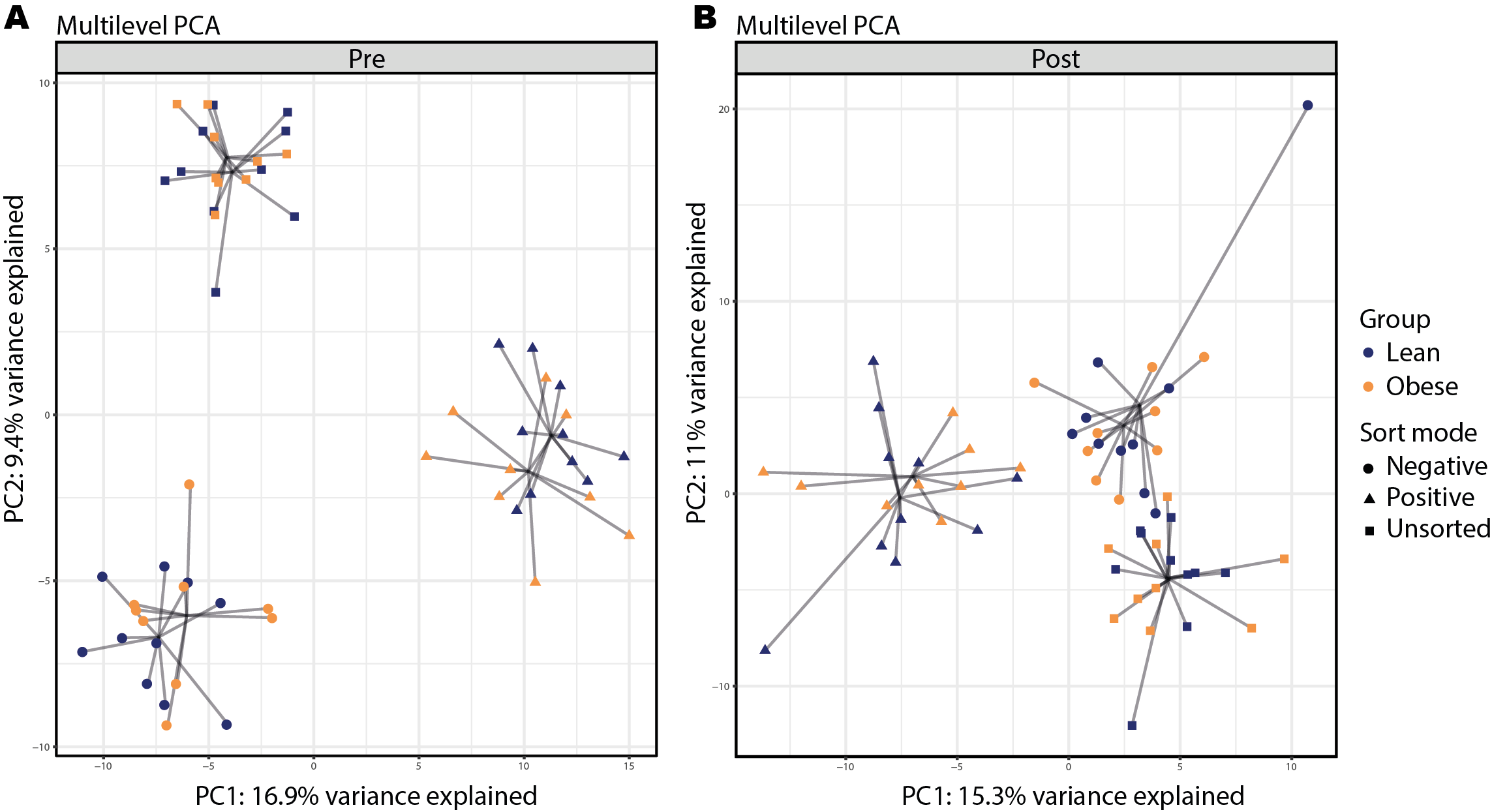


**Figure S2.** Principal component analysis (PCA) of IgA sorted bacteria.

Healthy lean (n = 10) or obese people with metabolic syndrome (n = 9) were given vancomycin for seven days. IgA-coated bacteria of the fecal microbiota were sorted and the bacterial 16S rRNA was sequenced.

**
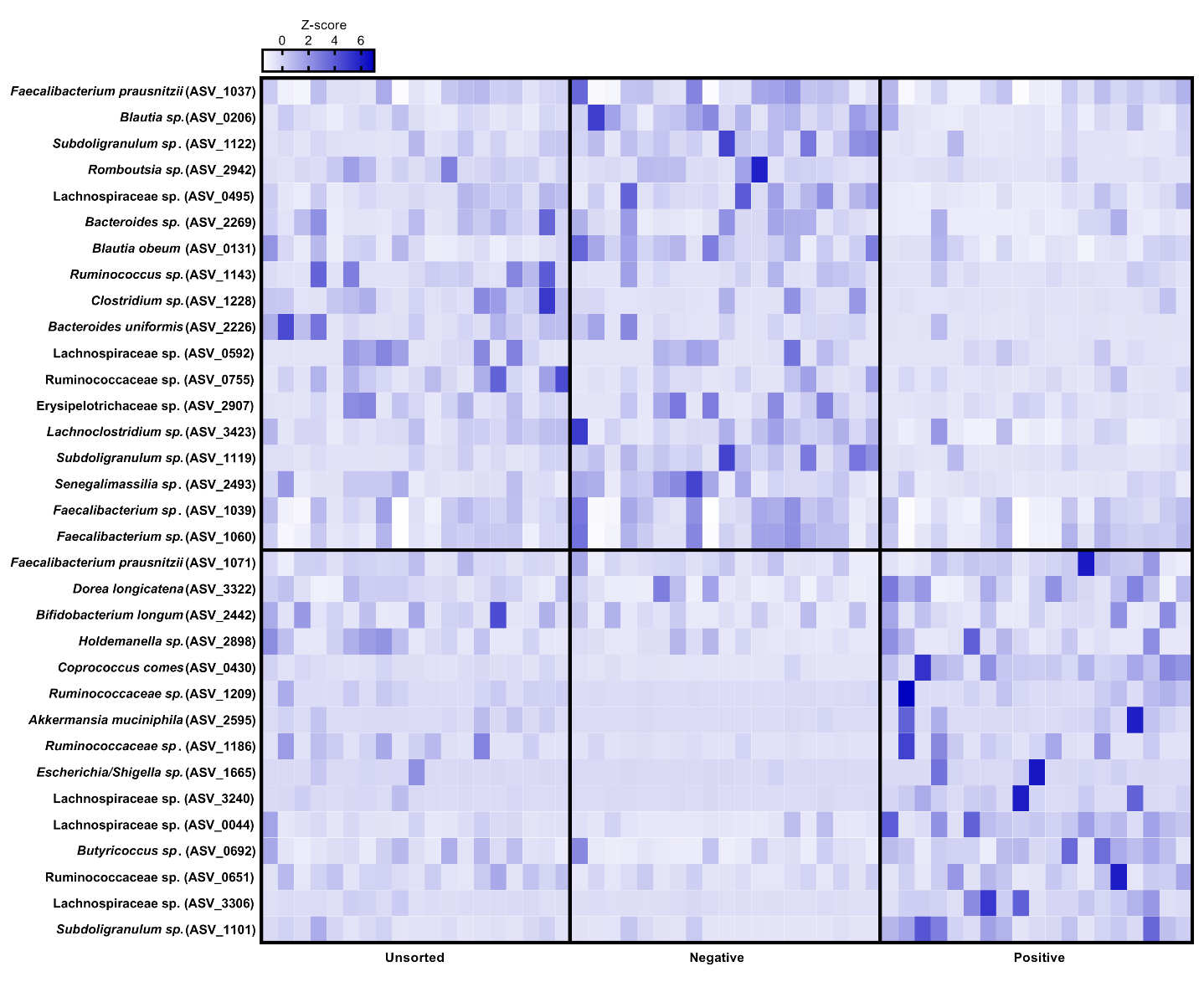
**

**Figure S3.** Differentially coated intestinal bacteria before vancomycin treatment.

Healthy lean (n = 10) and obese people with metabolic syndrome (n = 9) were given vancomycin for seven days. Intestinal bacteria were sorted and sequenced before treatment. The heatmap show the 30 most abundant bacteria (0.1%, at least present in 60% of the participants) that were significantly (p < 0.05) enriched either in the IgA negative (top) or positive (bottom) fraction. The heatmap was sorted according to their relative mean abundance (0.18% to 3.61%, bottom to top per fraction). Z-scores of their abundance are shown. Every column represents one participant. Statistical analysis was performed using Wilcoxon matched-pairs signed rank test.

**
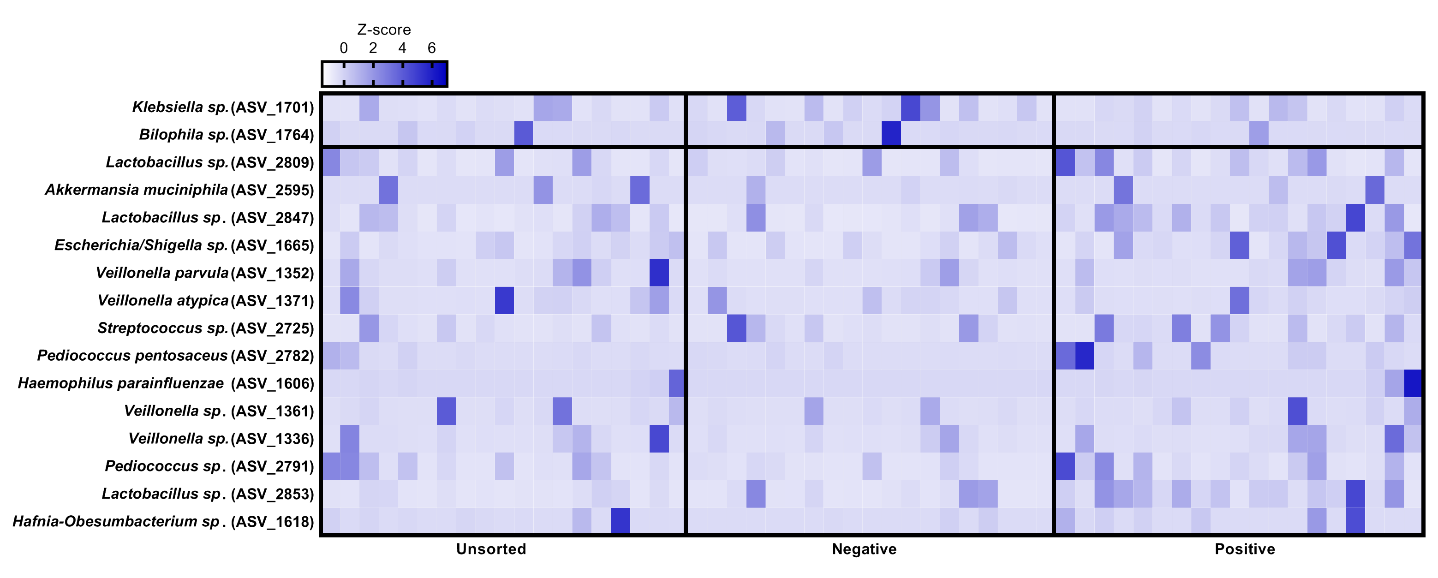
**

**Figure S4.** Differentially coated intestinal bacteria after vancomycin treatment.

Healthy lean and obese people with metabolic syndrome were given vancomycin for seven days. Intestinal bacteria were sorted and sequenced after treatment. The heatmap show the most abundant bacteria (0.1%, at least present in 60% of the participants) that are significantly (p < 0.05) enriched either in the IgA negative (top) or positive (bottom) fraction. The heatmap was sorted according to their relative mean abundance (0.14% to 6.59%, bottom to top per fraction). Z-scores of their abundance are shown. Every column represents one participant. Statistical analysis was performed using Wilcoxon matched-pairs signed rank test.


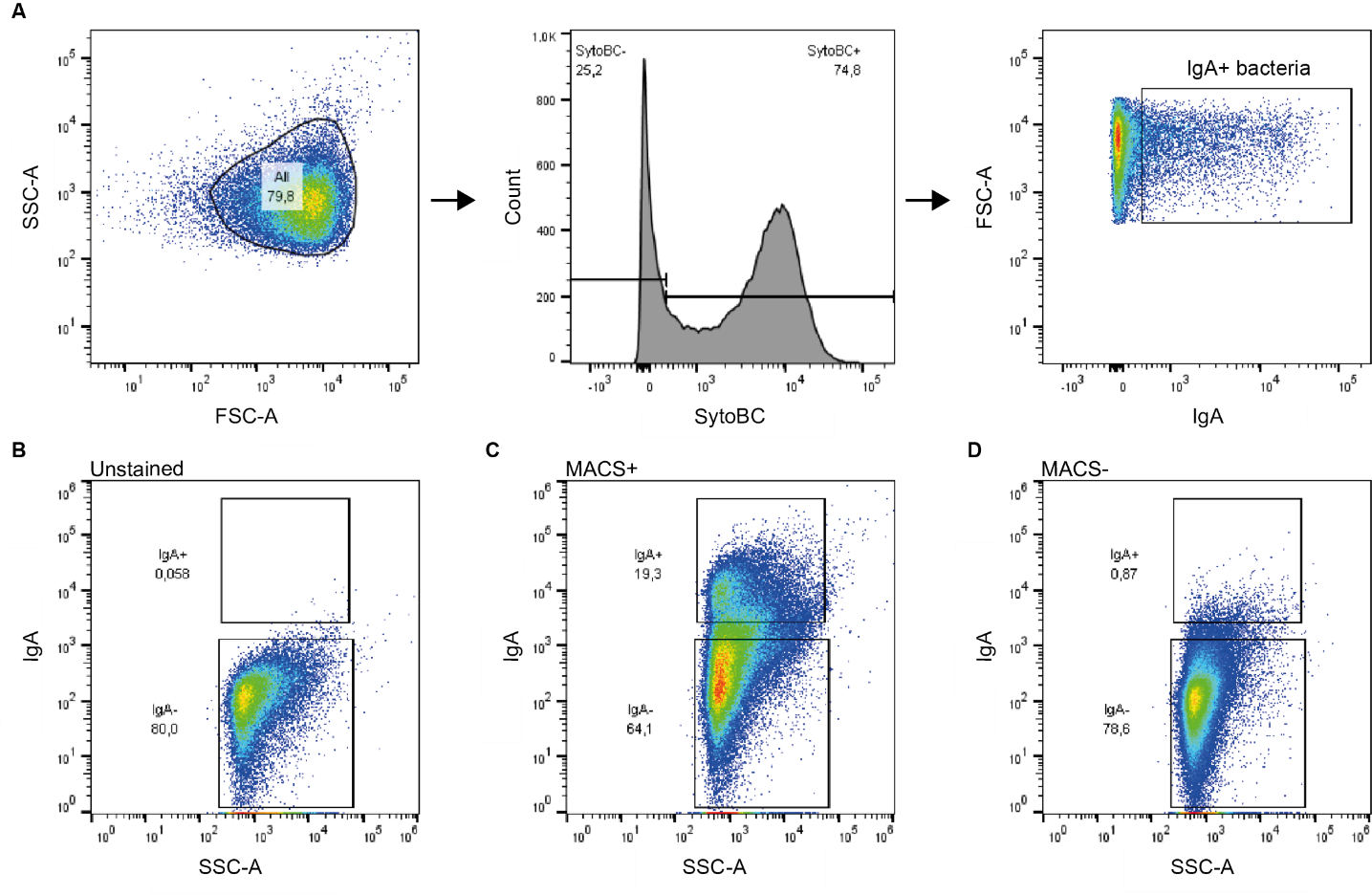


**Figure S5.** IgA analysis and sorting strategy.

Fecal samples were homogenized and stained with nucleic staining SytoBC (A) and an antibody against human IgA (A – D). For cell sorting, samples were enriched with magnetic beads (C, D). Samples were analysed on FACS Canto (A) and sorted on Sony sorter SH800 (B – D).

**Supplementary information**

**Figure S1. Serum antibodies do not change during high fat meal test.**

**Figure S2. Principal component analysis (PCA) of IgA sorted bacteria.**

**Figure S3.** **Differential coated intestinal bacteria before vancomycin treatment.**

**Figure S4. Differential coated intestinal bacteria after vancomycin treatment.**

**Figure S5. IgA analysis and sorting strategy.**

**Table S1. Characteristics of participants**

**Table S2. Antibody response during the mixed meal test.**

**Table S3. Combined LME analysis for IgA sorted bacteria.**

**Table S4. LME analysis before treatment.**

**Table S5. LME analysis after treatment.**
